# Molecular and microbiological methods for the identification of nonreplicating *Mycobacterium tuberculosis*

**DOI:** 10.1371/journal.ppat.1012595

**Published:** 2024-10-09

**Authors:** Jansy Passiflora Sarathy

**Affiliations:** 1 Center for Discovery and Innovation, Hackensack Meridian Health, Nutley, New Jersey, United States of America; 2 Hackensack Meridian School of Medicine, Department of Medical Sciences, Nutley, New Jersey, United States of America; University of Queensland, AUSTRALIA

## Abstract

Chronic tuberculosis (TB) disease, which requires months-long chemotherapy with multiple antibiotics, is defined by diverse pathological manifestations and bacterial phenotypes. Targeting drug-tolerant bacteria in the host is critical to achieving a faster and durable cure for TB. In order to facilitate this field of research, we need to consider the physiology of persistent MTB during infection, which is often associated with the nonreplicating (NR) state. However, the traditional approach to quantifying bacterial burden through colony enumeration alone only informs on the abundance of live bacilli at the time of sampling, and provides an incomplete picture of the replicative state of the pathogen and the extent to which bacterial replication is balanced by ongoing cell death. Modern approaches to profiling bacterial replication status provide a better understanding of inter- and intra-population dynamics under different culture conditions and in distinct host microenvironments. While some methods use molecular markers of DNA replication and cell division, other approaches take advantage of advances in the field of microfluidics and live-cell microscopy. Considerable effort has been made over the past few decades to develop preclinical in vivo models of TB infection and some are recognized for more closely recapitulating clinical disease pathology than others. Unique lesion compartments presenting different environmental conditions produce significant heterogeneity between *Mycobacterium tuberculosis* populations within the host. While cellular lesion compartments appear to be more permissive of ongoing bacterial replication, caseous foci are associated with the maintenance of *M*. *tuberculosis* in a state of static equilibrium. The accurate identification of nonreplicators and where they hide within the host have significant implications for the way novel chemotherapeutic agents and regimens are designed for persistent infections.

## Introduction

Antibiotic discovery is often focused on acute infections by pathogenic bacteria and overcoming genetic resistance. However, many persistent infections are difficult to treat for reasons other than gene mutations, leading to long-term chronic disease, treatment failure, and increased mortality [1,2]. Metabolic down-regulation, growth rate reduction, and the shift to a sessile lifestyle (planktonic versus biofilm states) are some of the major phenotypic changes associated with persistent bacterial infections [1,3]. In pulmonary tuberculosis (TB), patients presenting both subclinical and clinical forms of the disease, where the former present few or no signs and symptoms, macroscopic pathology progresses from cellular infiltration occurring at the site of failed *Mycobacterium tuberculosis* (MTB) containment [4]. Eventually, patients develop several lesion types with complex architectures and unique compositions [5–7]. Closed necrotic nodules and cavities contain caseous cores comprising of lipid-rich cellular debris [8,9]. Preclinical studies have demonstrated that caseous foci are refractory to antibiotic treatment, thereby necessitating long treatment durations [10–13]. Lesion-centric efficacy studies in rabbits and Cynomolgus macaques highlight deficiencies in self- (immune mediated-) and drug-mediated sterilization of caseous lesions and cavities compared to cellular lesions without central necrosis [14–16]. While intracellular MTB [17] and other infectious agents that cause protracted chronic infections [18,19] often rely on the manipulation of host cell responses to evade immune clearance, evidence suggests that the persistence of caseum MTB arises primarily from metabolic and physiologic adaptations to a hypoxic lipid-laden environment [20]. Common discourse describes caseum MTB as existing in the physiological state of nonreplication, in contrast to extracellular and intracellular MTB in other host compartments that maintain cell division at varying rates [7,20,21]. In the race to develop drugs that effectively target all bacterial populations within the host, thereby improving cure rates and shortening treatment duration, it has become increasingly critical to characterize the physiology and metabolic state of caseum MTB and develop “NR-active” antimicrobials [22–25].

One main challenge to the study of NR bacteria is the accurate profiling of the bacterial replication state to begin with. Historically, the enumeration of colony-forming units (CFUs) permitted the direct quantification of viable bacterial burden, and it is still the primary method used to profile mycobacterial growth kinetics in vitro and in vivo. However, in populations where net growth is absent or significantly reduced, colony counts alone do not reveal dynamic equilibrium scenarios with precisely balanced rates of cell replication and killing. This is a cause for concern in an age when in vitro models of nonreplicating persistence (NRP) are used routinely during TB drug discovery to screen for novel treatment-shortening agents and to decipher target essentiality [23,25–28]. The relative merits of the numerous in vitro NRP TB models that have been developed were explored extensively elsewhere [21,23,29–32]. Rather, this Review discusses the development of microbiological and molecular methods for the accurate characterization of NR bacterial populations within host organisms, with a focus on MTB and other mycobacterial species. Of note, the field has become increasingly reliant on modeling cumulative bacterial burden (CBB), which accounts for all live and dead bacteria in the axenic culture or infection system. This enumeration of all detectable bacteria regardless of viability offers a more accurate measure of MTB replication kinetics when active replication coincides with concurrent cell death [33–36]. This Review does not evaluate the nuanced definitions of “stationary-phase,” “dormant,” or “non-culturable” MTB, as was done previously [37–40], nor does it attempt to differentiate between “dormancy” and “persistence” [41,42]. Here, nonreplication simply refers to a culture in net growth stasis, in which overall bacterial replication is slowed dramatically or absent, but does not require that the population is metabolically inactive or exhibits phenotypic drug tolerance [23,33].

Specifically, we highlight 2 main discussion points for the methods presented here. Firstly, which techniques distinguish between NR bacteria and populations in dynamic equilibrium where replication is balanced by cell death? Secondly, how have these methods been applied to the study of MTB within various hosts and what was revealed about the influence of disease pathology on the pathogen’s replication status in the absence of antibiotic treatment? Many animal models of TB infection have been developed for the evaluation of vaccine and drug efficacy. While each model bears strengths and weaknesses, they present fundamental differences in pathological features and disease progression [43,44]. Murine TB infection models have been especially instrumental to the preclinical evaluation of novel drugs and drug regimens, but can produce profoundly different bacterial growth dynamics and treatment responses [45–48]. Across preclinical models, however, there is consensus in the definition of “chronic infection” as the progression to steady-state bacterial burden due to the onset of adaptive immune response [46,48–50]. This stasis is often interpreted as global replication retardation or arrest, regardless of the host organism or site of disease, but several lines of evidence point to ongoing MTB replication during chronic disease. Importantly, different lesion types within the same host can present different MTB phenotypes during natural infection, but information on inter-lesion and intra-lesion bacterial variability is often lost during gross lung homogenization. By evaluating multiple techniques that characterize bacterial replication status in vitro and in vivo, we consider how tuberculous lesion heterogeneity and caseous necrosis influence MTB replication dynamics.

## Colony-forming unit enumeration

One of the most rudimentary methods for characterizing growth- and kill-kinetics in bacterial cultures relies on simple CFU enumeration on solid medium. CFU counts are a direct measure of the viable bacterial burden in a given specimen. The staticity of CFU counts over time implies that the pathogen exists in a viable but NR state. This is typically how in vitro NRP MTB models are validated; growth arrest in oxygen-starved [51,52], nutrient-deprived [53], iron-starved [54], and streptomycin-dependent [55] models, to a name a few, was confirmed by observing stable CFU counts in the absence of increasing optical densities. Similarly, in vitro “persister” assays for the nontuberculous mycobacteria species *Mycobacterium abscessus* were validated by CFU enumeration on solid agar [56,57]. This technique, however, does not reflect the possibility that no net change in viable burden can represent a “dynamic equilibrium” between replication and cell death [33] (**Table 1**).

**Table 1:** Summary of methods for the comprehensive characterization of growth dynamics in mycobacterial populations.

Technique	Quantitative/qualitative analytical method	Distinguishes between NR bacteria and dynamic populations with no net growth	Population-wide observations	Single cell observations	Applicable to host tissue specimens	Ref.
Colony-forming unit enumeration	Colony growth on solid media	No	Yes	No	Yes	[53,56,57]
Acid-fast bacilli enumeration	Microscopy	Yes, when considered with CFU counts	Yes	No	Yes	[66]
Chromosome equivalents (CEQ)	Quantitative real-time PCR	Yes, when considered with CFU counts	Yes	No	Yes	[33]
Unstable plasmid replication clock	Quantitative real-time PCR	Yes	Yes	No	Yes	[34]
Fluorescent reporter systems for monitoring DNA replication and transcription	Confocal microscopy and live-cell time-lapse microscopy	Yes	Yes	Yes, with time-lapse microscopy	Yes	[76,77]
Microfluidics culture systems	Live-cell time-lapse microscopy in bright-field and fluorescent modes	Yes	Yes	Yes	Yes	[55,91]
Genomic sequencing read coverage	DNA sequencing	Yes	Yes	No	Yes	[92]
RS ratio	Droplet digital PCR and quantitative multiplexed RNA in situ hybridization (ISH)	Yes	Yes	Yes, with ISH	Yes	[94,95]

The chronic phase of infection in in vivo preclinical models is often defined by steady-state high bacillary loads that are quantified by CFU enumeration. The New Zealand White rabbit model of pulmonary TB, for instance, is characterized by dramatic growth in the early acute phase (4 weeks postinfection), followed by equilibration at high overall lung burdens until cavity formation at around 16 weeks postinfection may drive further localized bacillary growth [58,59]. Low- to moderate-dose aerosol infections of C57BL/6 and C3HeB/FeJ mice produce rapid pulmonary MTB growth during the first 2 to 4 weeks postinfection followed by sustained stable bacillary burdens as assessed by CFU enumeration too [12,34,60,61]. Similarly, low-dose *Mycobacterium marinum* infections in adult zebrafish (*Danio rerio*), another preclinical model that is applicable to the study of the wide spectrum of TB disease, produce early logarithmic bacterial growth followed by stable bacterial burdens (i.e., CFU per fish) and frequency of mature lesion formation from about 4 weeks postinfection [62–64].

In recent years, we have witnessed the major development of lesion-centric studies in rabbits [14,15] and nonhuman primates [11,16], where individual lesions are carefully excised and analyzed by traditional CFU enumeration, among other techniques, in order to build trajectories for replication dynamics and bacterial killing at these specific sites of disease. The measurement of “CFU per lesion” revealed that viable MTB burden in individual lesions from the same untreated animal can differ by several orders of magnitude even during steady-state chronic infections [14–16]. Furthermore, cavity caseum carefully explanted from infected rabbits and incubated ex vivo at 37°C presents no net increase in CFU burden, implying that the resident MTB population exists in an NR state [28,65]. Unfortunately, unchanging CFU abundance over time, whether derived from entire lung lobes or individual lesions, is insufficient to define populations in dynamic equilibrium. The rest of this Review considers techniques that should be used in addition to colony counting in order to properly characterize bacterial populations in “static equilibrium” (**Table 2**).

**Table 2:** Preclinical models of TB infection and the determination of bacterial replication dynamics in distinct host environments during chronic infection.

Host organism	*M*. *tuberculosis* strain	Necrotic lesion formation	Tissue compartment analyzed	Technique	Observations ^a^	Predominant replicative state of MTB during chronic infection ^b^	Ref.
BALB/c mouse	Erdman	No	Whole lung lobes	RS ratio	rRNA synthesis was reduced as CFU burden plateaued during chronic infection, but was far from maximally suppressed	Dynamic equilibrium	[94]
C57BL/6 mouse	H37Rv	No	Whole lung lobes	Unstable plasmid replication clock	Plasmid loss continued after CFU counts had stabilized; calculated cumulative bacterial burden increased throughout chronic infection	Dynamic equilibrium	[34]
	Erdman	No	Whole lung lobes	Chromosome equivalents	Minimal change in CEQ/CFU ratio during chronic phase of infection	Static equilibrium	[33]
C3HeB/FeJ mouse	Erdman	Yes	Cellular rim of necrotic lesions	RS ratio	Highly heterogeneous RS ratio measurements	Dynamic equilibrium	[94]
	Erdman	Yes	Caseum	RS ratio	Significantly lower pre-rRNA levels and inter-bacterial RS ratio heterogeneity in caseum compared to the cellular rim and remaining lung	Static equilibrium	[94–96]
	Erdman	Yes	Caseum	Single-stranded binding protein reporter strain (SSB-GFP)	Significantly more DNA replication detected in necrotic cores than in the macrophage phagosome-rich lesion cuff during the early stage of necrotic lesion formation	Unclear due to the limited comparison	[77]
New Zealand White rabbits	HN878	Yes	Cellular lesions without central necrosis	Chromosome equivalents	Gradual decline in median CEQ counts over the entire infection period	Unclear due to potential decay of CEQ	[14]
	HN878	Yes	Necrotic lesions	Chromosome equivalents	Consistent median CEQ counts during the chronic infection phase	Static equilibrium	[14,15]
	HN878	Yes	Caseum	Analysis of genomic sequencing read coverage	Sequencing reads displayed uniform coverage of the MTB genome with no peaks or troughs	Static equilibrium	[65]
Cynomolgus macaques	Erdman	Yes	Mixed lesions (caseous and non-necrotic)	Chromosome equivalents	Median CEQ counts in lesions in the early to advanced and latent stages of infection were similar	Static equilibrium	[16]

^a^ Observations from drug-naïve animals only.

^b^ “Static equilibrium” describes populations with slow and/or nonreplicating bacteria, whereas “dynamic equilibrium” describes populations where bacterial replication is balanced by killing at an almost equivalent rate [33].

### Acid-fast staining

In a seminal study in 1961, CFU counts defined using standard methods were compared to counts of microscopically detectable acid-fast bacilli (AFB) [66]. The ratio between the “viable population” and the “total stainable bacillary population” was established for lung specimens from TB-infected mice. The authors predicted that a dynamic scenario would present divergent CFU and AFB counts over time due to the accumulation of dead bacilli that would contribute only to the latter. By counting the number of stained bacilli in fixed-volume lung homogenate smears, they found that total AFB counts only slightly exceeded the viable counts in untreated mice 8 to 20 weeks postinfection. Both AFB and CFU counts in mouse lungs remained relatively stagnant at about 7.5 logs per animal. It was concluded that MTB in chronically infected mice exists in a static or resting state rather than in dynamic equilibrium between bacterial growth and death [66]. However, the loss in acid-fast staining of “dormant” MTB, which was observed in caseum and in a multi-stress in vitro NRP model [65,67], could have led to the significant underestimation of AFB/CFU ratios. Furthermore, this technique relies on the assumption that dead bacilli are not degraded over time. Clearance of dead bacilli by the host would also have resulted in the underestimation of CBB (live plus dead bacteria).

## Chromosome equivalents

Several molecular methods for the quantification of bacterial replication dynamics have been validated over the last 2 decades. One such method quantifies the number of MTB genome copies in host tissue as a measure of CBB. Also known as chromosome equivalents (CEQ), these copies were quantified by real-time quantitative PCR [33]. Specifically, primers and a molecular beacon were designed to amplify *fadE15* and quantify MTB chromosomal DNA in chronically infected C57BL/6 mice. This murine model only forms non-necrotic lesions during TB infection, where the pathogen remains intracellular [68]. Total CEQ counts (live and dead bacteria) were compared to CFU counts on agar (viable bacilli only). In C57BL/6 mice, CEQ/CFU ratios only changed minimally during the chronic phase of infection, indicating that bacterial replication reduced dramatically during this period. The lack of divergence between CFU and CEQ corresponded with the “static scenario” of host–pathogen equilibrium (**Table 2**) [33].

CEQ measurement was also used to assess the trajectory of MTB growth in cynomolgus macaques through the acute and chronic phases of infection [16]. TB pathology in this nonhuman primate model is highly similar to that of patients, with the formation of solid (cellular), caseous and cavitary pulmonary granulomas [50]. In that study, authors found that the medians of CEQ counts per lesion were stable over the different phases (acute, active, and latent) of TB infection (**Table 2**). CFU and CEQ data from the host as a whole best fit a logistic model in which there is a rapid growth phase postinfection, followed by drastically slowed bacterial growth coupled to immune-mediated cell death [16]. Lesions were purported to reach maximum bacterial burdens or “carrying capacities” with the onset of adaptive immunity, in line with the static equilibrium model defined earlier.

Similar efforts were made to profile the trajectory of bacterial burden in individual lesions from New Zealand White rabbits, another in vivo model that recapitulates key immuno-pathological features of TB in humans [58,59,69]. In those studies, cellular and necrotic pulmonary lesions were analyzed separately, allowing us to consider lesion-specific patterns of MTB growth and killing. In one study, median CEQ counts in drug-naïve necrotic lesions at 12 and 20 weeks postinfection (chronic phase) were similar to counts obtained after only 4 weeks [14]. In the other, there was no significant increase in median CEQ between necrotic lesions collected from drug-naïve rabbits at 12 and 16 weeks postinfection [15]. Taken together, these results also indicate that necrotizing TB pathology supports static NR bacterial populations (**Table 2**). This was further emphasized by the observation of excised rabbit caseum incubated under ex vivo growth-permitting conditions; CEQ/CFU ratios remained constant in the absence of antibiotic treatment [65]. CEQ measurements rely on the assumption that chromosomal DNA is not degraded by the host over time, which would result in the underestimation of CBB. The decline in CEQ per lesion in rabbit cellular granulomas over weeks of infection indirectly points to gradual decay of MTB DNA within the host, likely by activated immune cells [14]. However, the rate of MTB genomic decay in macaque lesions was found to be slow enough to permit the use of CEQ to estimate CBB [16].

## Unstable plasmid replication clock

In an effort to build a “replication clock” to study MTB replication dynamics in vitro and in vivo, one group validated the use of an unstable plasmid that is lost at a steady quantifiable rate in growing cultures [34]. The circular plasmid pBP10 carries a kanamycin resistance marker that replicates consistently with MTB chromosomal DNA in the presence of the selection antibiotic. In the absence of kanamycin, the plasmid is lost from a fixed proportion of daughter cells during cell division, regardless of the replication rate. The authors modeled the segregation constant *s*, or the frequency of plasmid loss in daughter cells per generation, in MTB cultures under different growth-permitting and -limiting in vitro conditions. They found that oxygen- and carbon-starvation, which produce NR drug-tolerant MTB cultures, resulted in no plasmid loss in surviving cells (**Table 1**). Stationary-phase cultures grown for about 20 days without antibiotics or subculturing also displayed no plasmid loss after stable CFUs were achieved [34]. Accordingly, a subsequent study used the replication clock plasmid to validate an iron-starvation in vitro NRP MTB model [54].

In contrast, untreated C57BL/6 mice with chronic TB infections displayed steady plasmid loss even after the stabilization of CFU counts. The segregation constant was used to calculate the growth rate, death rate and CBB for different time intervals during the whole infection period. While CFU counts stabilized after 4 weeks of infection, CBB continued to grow to 10-fold greater than CFU counts by week 16. Calculated MTB generation times were 21.4 h and 96 h during the first 2 weeks and at week 16, respectively, indicating slowed but still significant bacterial growth in the host during the chronic phase of infection [34]. Altogether, these results suggest that static CFU counts produced in this non-necrotizing murine model actually represents an equilibrium between MTB replication and killing (**Table 2**). The authors noted, however, that their population-wide observations did not distinguish between the possibility of slowed but continuous replication of the entire pathogen population and the possibility of a subpopulation of rapidly dividing MTB among nonreplicators. The replication clock plasmid has a wide variety of applications, which includes the elucidation of cell replication defects of mutant strains in mouse infection models in order to demonstrate the essentiality of specific target genes [36,70]. pBP10 was used to study MTB growth and death rates in murine bone marrow-derived macrophages in the absence of drug treatment. The pBP10 plasmid loss profile and corresponding CFU counts indicated rapid MTB replication coupled to even greater killing during the first 2 days postinfection, followed by an extended phase of much slower replication coinciding with increased viability [71]. Through the isolation and sorting of different phagocyte populations from MTB Erdman-pBP10–infected mice, it was also revealed that interstitial macrophages are more capable of limiting growth and promoting killing of intracellular bacilli than alveolar macrophages during acute infection [72].

### Fluorescent reporter systems for monitoring DNA replication and transcription

Much progress has been made in the use of fluorescent-tagged components of the replisome to track the cell cycle of replicating bacteria. The single-stranded binding protein (SSB), for instance, ubiquitous in prokaryotes and eukaryotes, binds DNA and maintains genome integrity during replication, recombination, and repair [73]. Reporter strains have been engineered by fusing *ssb* to green fluorescent protein (GFP) on a replicating plasmid containing a constitutively expressed mCherry. SSB-fluorophore fusions have been validated extensively as markers of active DNA replication in *Escherichia coli*, *Bacillus subtilis*, and *Mycobacterium smegmatis* [74–76]. Fluorescence of the replication focus marks the initiation and the entire duration of DNA replication. Foci then disappear upon termination of DNA replication, thereby defining cell cycle timing in growing bacteria.

The dynamics of SSB-GFP expression in mycobacterial strains were established using live-cell time-lapse microscopy. In a logarithmic-phase broth culture of MTB, up to 80% of the bacilli presented the SSB-GFP foci. Importantly, the SSB-GFP foci disappeared in non-growing stationary-phase cultures [76]. The Erdman(SSB-GFP, smyc′::mCherry) reporter strain has been used to visualize heterogeneity in replication status of MTB in vaccinated versus mock-treated mice [76], and of intracellular MTB in different phagocyte populations [72]. It is important to note that the lack of an SSB-foci, however, does not distinguish between an NR bacterium and a replicating one outside the cell cycle stage of DNA replication. Nevertheless, reporters of the replisome focus can provide spatial information about the replication status of individual bacilli in host microenvironments (**Table 1**). To this end, Lavin and Tan performed targeted 3D imaging of large Type I (caseous) lesions from untreated MTB-infected C3HeB/FeJ mice, with a broad xy-plane tiled imaging approach to provide the breadth required to capture these lesions in their entirety. A greater percentage of MTB in the necrotic core presented the SSB-GFP foci, compared to the macrophage phagosome-rich lesion cuff where low pH is believed to significantly limit bacterial replication (**Table 2**). Hence, this methodology provides the framework for in situ analysis of intra-lesion MTB heterogeneity at the single cell level [77].

Alternative fluorescent reporter systems that highlight chromosomal replication could be applied to the study of NR MTB. Several studies have successfully used DnaN-fluorescent protein to mark sliding clamps and help visualize DNA replication forks in *E*. *coli*, *B*. *subtilis*, and *M*. *smegmatis* [78,79]. Dual reporter mycobacterial strains that express Wag31-GFP and mCherry-DnaN mark the appearance of the cell division septum and the DNA replisome complex, respectively, during time-lapse microscopy [80]. On another note, replicating cells invest significant energy and metabolic resources in protein synthesis. Hence, transcriptional reporter strains have also been useful at profiling the NR state. *E*. *coli* strains were engineered to carry plasmids with the promoter regions of genes that are up-regulated during persistence (i.e., *tolC* and *tnaC*) inserted upstream of a gene for fast-folding GFP to investigate replication dynamics and antibiotic susceptibility in NR cultures [81]. Similarly, GFP-tagged ribosomal RNA (rRNA-GFP) has permitted the tracking of transcriptional activity and MTB growth dynamics in vitro and in infected mice [82]. Switching from complete growth-permitting broth medium to phosphate-buffered saline (PBS) to create an extended (3 months) nutrient-starved stationary phase culture resulted in a rapid decline in rRNA-GFP fluorescence and subsequent growth arrest. Furthermore, rRNA-GFP-expressing MTB explanted from C57BL/6 mouse lungs displayed progressive reductions in reporter fluorescence and regrowth potential over the course of the 16-week acute-to-chronic infection, reflecting on the impact of host immunity alone on bacterial physiology and growth kinetics [82].

### Microfluidics-based culture systems

Microfluidics systems coupled to time-lapse microscopy have been critical to the study of inter-bacterial heterogeneity in vitro. Microfluidics platforms that probe antibiotic dose-response are especially instrumental to the elucidation of pharmacokinetic-pharmacodynamic (PK-PD) relationships in space and time [83]. These systems differ in the way microfluidic chips are structured and fabricated, the way and rate at which fluids are moved through micro-channels, and the analytical software used for lineage tracking [84,85]. Nevertheless, they operate on the fundamental principle that microfluidics confinement and time-lapse microscopy permit the tracking of individual bacterium (**Table 1**). Bright-field images, and corresponding fluorescent images for live/dead staining, provide quantitative information about the width, length, area, and fluorescence intensity of single cells as well any resulting progeny. Using such technology, it was revealed that elongation and cell division in *Mycobacterium smegmatis* and MTB is significantly less symmetric than in *E*. *coli*, contributing to daughter cells that differ in elongation rate and size [86]. In another study, authors were able to distinguish between 2 distinct growth-arrested phenotypes in *E*. *coli*; persistent bacilli that survive antibiotic exposure but resume replication upon removal of the antibiotic, and viable but non-culturable (VBNC) cells that remain NR for prolonged periods of time despite the return of growth-permitting conditions [81]. Importantly, microfluidics systems revealed that persistence in the face of antibiotic exposure is linked to inherent heterogeneity in bacterial populations, as cells switch between normal and reduced growth rate phenotypes even in the absence of treatment, possibly as an adaptation to fluctuating environmental conditions during infection [87].

Where mycobacterial species are concerned, time-resolved bright-field imaging of single cells and microcolonies has also been made possible with microfluidic technology [83,88,89]. This technique was used to demonstrate replication-arrest of the streptomycin-dependent 18b MTB strain upon the withdrawal of the antibiotic. Thousands of individual mycobacterial cells were monitored for elongation and division during STR starvation to validate the in vitro NRP model [55]. Alternatively, GFP-expressing mycobacterial strains have been observed on microfluidics devices with the aid of fluorescence or confocal laser scanning microscopy [81,82,88,90]. The relatively small size, nonspherical shape and 3D clustered growth of mycobacterial species make it especially challenging to track single bacterium replication in long-term cultures [88]. For instance, one such platform permitted single-cell observation of *M*. *smegmatis* up to 5 generations, at which point images became too dense to score [86]. Several approaches to confining mycobacterial growth on a single focal plane, such as the addition of an alginate hydrogel matrix [90], have improved real-time analysis. Staining with propidium iodide facilitates the simultaneous detection of cell death [81,88,90]. Microfluidics can also cater to the observation of intact live tissue microenvironments on a sub-millimeter scale [91], although this application is not yet evident in the field of TB research (**Table 1**).

### Genomic sequencing read coverage

A new metagenomic data analysis method was developed to measure growth dynamics of individual bacterial species in complex microbiota communities using single snapshot samples [92]. Most bacterial species carry a single circular chromosome and display bidirectional replication from a fixed point of origin toward a single terminus [93]. During DNA replication, regions of the chromosome that have been passed by replication forks will have 2 or more copies, unlike yet unreplicated regions near the terminus which are present as a single copy. Each bacterial cell in a dynamic population was captured at a different stage of DNA replication, contributing to a coverage pattern for the entire population that peaks at the replication origin [92]. The resultant pattern of sequencing read coverage from a culture experiencing exponential growth had a single peak and a single trough.

Importantly, non-dividing bacterial cells typically display single copies of the chromosome. Hence, NR populations produced flat sequencing coverage patterns across the genome. This phenomenon was initially demonstrated with an in vitro *E*. *coli* stationary phase cultures [92]. This technique was also used to characterize native MTB in rabbit caseum specimens. Normalized genome coverage of intra-caseum MTB presented the flat genome sequencing read pattern typical of NR bacteria, even during ex vivo incubation under growth-permitting conditions (**Table 2**) [28]. In the same study, this molecular technique was used to demonstrate that once actively replicating MTB from broth culture was inoculated into caseum surrogate, a foamy macrophage-based matrix, the bacteria population displayed increasing uniformity of genome coverage over time. The experiment helped validate the use of caseum surrogate in a novel in vitro NRP MTB model [28].

In the original publication of this method, the authors used the ratio of sequencing coverage between the origin of replication and the terminus, which was labeled the peak-to-trough ratio (PTR), to provide a quantitative estimate of the growth rate of a bacterial population. PTR correlated well with optical density-based growth rate measurements in *E*. *coli* cultures. The relationship between PTR and growth rate extended to other commensal strains and to growth-restricted conditions as well. PTR predicted reductions in *Lactobacillus gasseri* and *Enterococcus faecalis* growth rates brought about by shifts from anaerobic to aerobic culture conditions [92]. Overall, PTR measurements are capable of deciphering microbial kinetics in complex microbiome populations with mixed bacterial communities, setting it apart from most of the molecular techniques presented here.

### RS ratio

Ribosomal RNA (rRNA) synthesis is a fundamental physiological process that is being used as a pharmacodynamic marker of bacterial growth and antibiotic sterilizing activity. Premature rRNA in MTB include mature rRNA segments (16s and 23s) and short-lived spacer sequences. The latter can be further identified as external transcribed spacer 1 (ETS1) and internal transcribed spacer 1 (ITS1), depending on their positioning on the operon [94]. Both spacer types are rapidly degraded, leaving behind the stable mature rRNA. As a result, the relative abundance of pre-rRNA and mature rRNA provides a measure of ongoing rRNA synthesis in bacterial cells, and a surrogate measure of growth (**Table 1**). The rRNA synthesis (RS) ratio, defined as the ratio of ETS1 to 23S rRNA copies quantified by droplet digital PCR, was used to study bacterial replication dynamics in the oxygen depletion model of NRP TB and in the lungs of TB-infected BALB/c mice. During in vitro oxygen depletion, the gradual decline in RS ratio mirrored the decrease in MTB growth rate [94]. Much like C57BL/6 mice, the BALB/c mouse model does not produce necrotizing lesions [68]. Interestingly, the RS ratio in this murine TB model decreased gradually during the course on the chronic infection but was never maximally suppressed to levels observed in oxygen-starved in vitro cultures. Hence, the plateau in CFU burden produced in untreated BALB/c mice after 4 weeks of infection is indicative of a dynamic balance between MTB replication and death rather than a true NR population (**Table 2**) [94].

Additionally, quantitative multiplexed RNA in situ hybridization (ISH) was used to visualize and quantify the relative abundance of pre-rRNA to 23S signals in individual MTB bacilli in thin sections of C3HeB/FeJ mouse lung granulomas. Unlike BALB/c mice, C3HeB/FeJ mice develop granulomas with central necrosis containing an abundance of extracellular bacilli [61,68]. Pre-rRNA ISH indicated significantly lower MTB rRNA synthesis in central caseous compartments than in immune cell-rich inflammatory rims [94,95]. Importantly, low RS ratios in caseum with little heterogeneity between individual bacilli are consistent with the NR state (**Table 2**) [94]. A follow-up study in drug-naïve C3HeB/FeJ mice further confirmed that caseous microenvironments host large MTB populations with very low rates of ongoing rRNA synthesis, thereby also establishing that higher CFU counts are not necessarily associated with higher RS ratios [96].

## Discussion

Persistent bacterial infections, even in the absence of antibiotic resistance, often require aggressive combination chemotherapy and prolonged treatment durations. During pulmonary TB, MTB occupies diverse microenvironments in the host, each presenting unique stress factors and host–pathogen interaction interfaces, resulting in phenotypically heterogeneous subpopulations [21,97]. The occurrence of drug tolerant MTB populations in caseous lesion compartments during natural infection prior to chemotherapy increases chances of treatment failure and relapse [20,65]. As the TB research community continues to grapple with the concept of NR populations in the host, it is critical that we recognize that the observation of population-wide growth stasis in vitro or in vivo can arise from an equilibrium between replication and killing. CFU enumeration remains the method of choice for the quantification of overall bacterial burden and growth kinetics but is insufficient to reveal bacterial replication masked by ongoing cell death. The development of newer molecular and microscopic techniques has enabled more accurate characterization of bacterial replication rates within host organisms, and highlights key facets of MTB physiology during infection. Some of the methods described here, such as CEQ measurement and the use of an unstable plasmid, enable CBB quantification and successfully highlight bacterial replication in apparently stationary populations. However, such methods do not permit the observation of single cells and inter-bacterial differences in replication rate (**Table 1**). As a result, they are not suitable to distinguish between a global reduction in MTB replication and the possibility that a subset of bacteria maintain active replication amid a largely NR population. Indeed, several lines of evidence suggest that persistent bacilli preexist in all culture systems and in vivo environments, albeit in varying proportions of the overall burden [42,87]. Real-time live-cell imaging coupled to microfluidics culture systems or specific fluorescent reporter strains overcome the challenges of studying inter-bacterial differences and continue to unravel the complex dynamics of mycobacterial replication under diverse culture conditions and in host–pathogen interaction platforms [76,80,98–100].

C3HeB/FeJ mice, rabbits, guinea pigs, and marmosets, to name a few host species, form necrotic lesions with caseous cores subtended by cellular rims, similar to lesions developed during clinical TB [5,48,101]. Caseum in particular is avascular and mostly acellular, further characterized by low oxygen tension and high lipid content [20]. These stress conditions are critical to the activation of intrinsic pathways in MTB related to the down-regulation of metabolic activity and the acquisition of phenotypic antibiotic resistance. The type of murine preclinical model employed during preclinical efficacy studies can have significant influence on the interpretation of drug and vaccine efficacy. As discussed above, the C3HeB/FeJ mouse model is unique in its presentation of caseous lesions among other non-necrotic lesion types [61], and studies have shown that this translates to poorer treatment response [12,102,103]. Immune responses and bacterial phenotype aside, differences in composition and architecture between cellular and necrotic lesions produce variability in drug distribution as well, further compromising lesion sterilization in the latter [102,104,105].

Overall, any host model that presents a variety of lesion types holds mixed MTB subpopulations with varying metabolic and replication rates. Multiple studies have demonstrated that non-necrotic cellular lesions support ongoing MTB replication. Only CEQ measurement in C57BL/6 mice provided contradicting findings [33] (**Table 2**), most likely due to the degradation of chromosomal DNA from dead MTB, as observed in rabbit cellular lesions [14], which confounds the interpretation of CBB. Preclinical findings also point to a strong association between caseating necrosis and MTB replication arrest (**Table 2**), with the exception of a study which observed relative SSB-GFP reporter signals during the early stages of necrotic lesion formation in C3HeB/FeJ mice [77]. Admittedly, those experiments compared caseum to phagosome-rich lesion cuffs, where acidic pH limits MTB replication, rather than growing populations. Longer-term infections with more stable reporters could reveal how the kinetics of MTB DNA replication evolves with time and infection severity. Altogether, we should consider that the fate of individual lesions can vary substantially. Higher animal TB infection models such as the cynomolgus macaque produce caseous and non-necrotic lesions with diverse and overlapping bacterial burden trajectories, suggesting that critical host responses at the lesional level ultimately determine local bacterial control and overall clinical outcome [16]. Single-cell mRNA sequencing of individual TB lung granulomas from infected macaques revealed the association between different functional clusters of multiple immune and non-immune host cells and the extent of bacterial burden control at the lesional level [106]. Also, the emergence of a handful of necrotic lesions with extremely high CBB (10^7^ to 10^9^ CEQ per lesion) in rabbits during late-stage TB infection (>16 weeks) is a possible indicator of the resumption of bacterial replication during lesion cavitation and the erosion of necrotic centers [14,15], in agreement with high burdens of viable MTB occasionally observed in human cavities [6,107,108], although more work needs to be done to investigate this phenomenon. Ultimately, it is worth recognizing 2 key concepts about mycobacterial ecology in caseous foci in both closed and cavitary lesions. (1) While caseum presents growth-limiting conditions that induce MTB to stagnate and persist for long durations, its composition and consistency can evolve during cavitation such that more growth-permissive characteristics enable the reactivation of replication and disease relapse [108–110]. (2) Oxygen and nutrient gradients present throughout zones of necrosis, dependent on proximity to vasculature and lumens, most likely lead to the 3D organization of MTB in varying metabolic and transcriptional states [108].

The use of whole genome sequencing to estimate the mutation rate of MTB in drug-naïve cynomolgus macaques with active, latent, and reactivate disease revealed that the pathogen accumulates chromosomal mutations during latency at the same rate as during active infection and in logarithmic growing broth cultures. These findings could have suggested that MTB maintains active replication throughout the course of subclinical disease, also referred to as latent infection. However, the study also revealed that the polymorphisms identified in MTB isolates from macaques with persistent latency resemble products of oxidative damage rather than replicative error, consistent with findings of a highly oxidative environment in macrophage phagolysosomes. Therefore, the mutational capacity of MTB during latency in nonhuman primates is most likely the result of immune-mediate DNA damage instead of the byproduct of rapid bacterial replication [111]. While this Review has presented multiple lines of evidence for the NR phenotype in necrotic foci during natural infection, it has not addressed the distinct phenomenon of drug-induced growth arrest. Of note, isoniazid treatment of axenic MTB expressing rRNA-GFP resulted in the identification of a non-growing but metabolically active (NGMA) survivor population. The prominence of NGMA bacteria in isoniazid-treated mice suggests a role in TB persistence and disease relapse [82].

Many in vitro NRP models have been developed to generate nonreplicating persistent (NRP) MTB cultures. They use environmental stresses such as oxygen deprivation, nutrient starvation, low pH, and reactive nitrogen species to reproduce diverse microenvironments within the host [21,23,29–32]. Interestingly, intracellular lipophilic inclusions (ILI), spherical lipid storage vesicles present in many bacterial species, appear to represent another physiological marker of nonreplication in MTB. ILI have been detected in caseum MTB [28,65], clinical sputum specimens [112,113], MTB in foamy macrophages [114,115], and several NRP models [28,116,117], in contrast to replicating cultures which present minimal intracellular lipid storage [28,65,113]. Furthermore, the reversal of ILI accumulation leads to the resumption of mycobacterial growth [118]. Mycobacterial ILI are rich in triglycerides, polar lipids, and wax esters, thereby serving as long-term storage of carbon-based energy [20,117,119,120]. Additional investigation is required to determine whether ILI are a universal feature of all NR MTB cultures regardless of environmental stimuli, allowing them to be used as yet another marker of the NR state.

The techniques presented in this Review were discussed mainly with respect to MTB but can be applied to any bacterial species. Apart from the search for nonreplicators, CEQ and RS ratio are being used as pharmacodynamic markers of lesion sterilization potential and treatment-shortening activity in preclinical drug efficacy studies [14,15,94]. In summary, many advances have been made in the development of molecular tools that reveal in vitro and in vivo bacterial replication dynamics at the population and single-cell levels. Apparent growth stasis during chronic TB infection in any host is often the net effect of bacterial replication and immunological killing, and evidence suggests that NR MTB subpopulations are mostly limited to zones of caseous necrosis. New and improved techniques for the evaluation of NR bacteria will facilitate the development of better diagnostic markers and sustainable treatment options for TB and other persistent infections.
